# Supplementary material for: Translating Biomarkers of Cholangiocarcinoma for Theranosis: A Systematic Review
Source: Cancers (Basel). 2020 Sep 30;12(10):2817. doi: 10.3390/cancers12102817 (PMC7601719; doi:10.3390/cancers12102817)
Supplement: Supplementary file 1 [file cancers-12-02817-s001.pdf]

Supplementary Materials: Translating Biomarkers of Cholangiocarcinoma for Theranosis: A Systematic Review

Imeshi Wijetunga, Laura E. McVeigh, Antonia Charalambous, Agne Antanaviciute, Ian M. Carr, Amit Nair, K. Raj Prasad, Nicola Ingram and P. Louise Coletta

Table 1. Data extraction table. A summary of the 166 candidate biomarkers that met the inclusion/exclusion criteria. List of acronyms and abbreviations used: BD = bile duct, CCA = cholangiocarcinoma, i/e/pCCA = intrahepatic/extrahepatic/perihilar CCA, CCC = cholangiocellular carcinoma, NR = Not Reported, occ = occasionally, % + = percentage positive (+ve), PSC = primary sclerosing cholangitis, TMA = tissue microarray, unspec = unspecified.

AA Biomarker (*Gene*)	Year	First Author PMID	Eastern Study?	Site of Tumor	No. of Patients	% up-Regulation	% +	Location of Marker	Slide/ TMA	Control Group	Expression in Normal Tissues	Risk of Bias
A1AT *SERPINA1*	2013	Jamnongkan, W. 23188705	Thailand	CCA	42	57.1%	-	Cytoplasm	S	Inflammatory tissues	Weak +ve normal BD	NR
A1AT *SERPINA1*	2017	Laohaviroj, M. (2017) 28618946	Thailand	CCA unspec	354	NR	50%	Cytoplasm	TMA	*n* = 2 normal tissues	NR	2 blinded indepen dent
A1AT *SERPINA1*	2014	Khenjanta, C. 25556446	Thailand	iCCA	30	23% (7/30)	NR	Tumour cells	S	*n* = 30, adjacent normal tissue	Weak staining	2 blinded indepen dent observer s
AFP *AFP*	2017	Liu, S. 28915621	China	iCCA	60	-	53.3 %	NR	S	*n* = 60 paired normal tissue	NR	NR
Akirin2 *AKIRIN2*	2019	Leng, K. 30886152	China	CCA	51	30/51	NR	Nucleus	S	Paired non-tumour *n* = 51	Normal tissue low	NR
Ang1 *ANGPT1*	2008	Mihara, Y. -	Japan	eCCA	119	-	43.7 %	Cytoplasm	S	Normal mucosa *n* = 38	15.8%	2 indepen dent investiga tors
Ang1 *ANGPT1*	2006	Tang, D. 16465407	Japan	CCA (CCC)	33	15.2%	42.4 %	Cytoplasm	S	NR	Hepatocytes of adjacent liver tissue	NR
Ang2 *ANGPT2*	2008	Mihara, Y. -	Japan	eCCA	119	-	42.0 %	Cytoplasm	S	Normal mucosa *n* = 38	26.3%	2 indepen dent

												investiga tors
Ang2 *ANGPT2*	2006	Tang, D. 16465407	Japan	CCA (CCC)	33	15.2%	57.6 %	Cytoplasm	S	NR	Hepatocytes of adjacent liver tissue	NR
Ang2 *ANGPT2*	2014	Voigtlander 24823366	No, Germany	eCCA iCCA	37 8	Strong induction in tumour vasculature	NR	Tumour vascular endothelium	S	PSC	PSC negative.	NR
Aromatase *CYP19A1*	2018	Kaewlert, W. 30284180	Thailand	CCA	74	61%	-	Cytoplasm	S	Normal bile duct cells	Lower than CCA	NR
CA9 CA9	2019	Bi, C. 30849962	China	iCCA	113	85.0%	-	NR	S	NR	NR	2 indepen dent patholog ists
CA9 *CA9*	2014	Gu, M. 25755787	Korea	iCCA	85	NR	44.7 %	NR	TMA	n = 4 normal liver tissue	NR	NR
Cadherin 17 *CDH17*	2015	Maeda, S. 25917007	Japan	eCCA	165	-	41%	NR	S	NR	NR	3 observer s
CD24 *CD24*	2006	Su, M.-C. 16125303	Taiwan	iCCA	70	NR	51%	Membrane Cytoplasm	S	Normal liver tissue	-ve	NR
CD24 *CD24*	2013	Kim, K. 23539485	Korea	eCCA	84	NR	42.9 %	Tumour cells	TMA	None	NR	NR
CD24 *CD24*	2010	Riener, M.-O. 20621328	Switzerlan d	iCCA eCCA	19 59	NR	21% 58%	Cytoplasm	TMA	n = 30 normal biliary epithelium of extrahepatic BD	Negative for CD24 expression	NR
CDX2 *CDX2*	2018	Tang, H. 29862152	China	iCCA	93	-	29.0 %	Nucleus	S	NR	NR	NR
CDX2 *CDX2*	2018	Wang, Z. No PubMed ID	Japan	iCCA	127	-	26.0 %	Nucleus	TMA	NR	NR	2 blinded observer s

CDX2 *CDX2*	2019	Ishida, K. 31401234	Japan	eCCA	84	NR	NR	Nucleus	S	NR	NR	3 blinded independent observers
CDX2 *CDX2*	2007	Chang, Y. 17295772	Thailand	eCCA	49	-	22.5 %	Nucleus	S	NR	NR	Single independent pathologist
CDX2 *CDX2*	2006	Jinawath, A. 16794828	Thailand	iCCA eCCA	36 23	-	37.4 %	NR	S	NR	NR	NR
CDX2 *CDX2*	2008	Chiu, C.-T. 18329969	Taiwan	iCCA	30	NR	60%	Nucleus	S	None	NR	NR
CDX2 *CDX2*	2017	Hass, H.G.	Germany	iCCA	36	NR	25%	Nucleus	S	NR	NR	NR
Claudin 18 *CLDN18*	2015	Keira, Y. 25503275	Japan	iCCA eCCA	27 32	NR	NR	Basolateral membrane	S	NR	non-neoplastic cells -ve	NR
Claudin 18 *CLDN18*	2011	Shinozaki, A. 21607649	Japan	eCCA iCCA	99 83	NR	90% 43%	Membrane	S	Normal intrahepatic and extrahepatic biliary epithelial	-ve. Inflammatory mucosa basolateral membrane occ +ve	NR
CTLA4 *CTLA4*	2017	Lim, Y.J. 28084572	Korea	eCCA	77	79.2% (61/77)	NR	Tumour cells and infiltrating lymphocytes	TMA	None	NR	2 blinded independent observers confirmed the results
CUL4A *CUL4A*	2017	Huang, G.-K.	Taiwan	iCCA	105	NR	32.4 %	Nucleus	TMA	Normal BD	NR	Two blinded

		28576144									independent observers
CUL4A *CUL4A*	2017	Zhang, T.-J. 28428711	China	pCCA	72	NR	NR	Nucleus	S	*n* = 12 adjacent normal BD from the 72 patients	Expressed in normal BD	Two blinded independent observers
CXCL12 *CXCL12*	2019	Miyata, T. 31361379	Japan	iCCA	127	52% (66/127)	NR	Nucleus and cytoplasm	S	NR	NR	Two blinded independent observers
CXCR2 *CXCR2*	2014	Sueoka, H. 24582495	Japan	iCCA	34	64.7% (22/34)	NR	Tumour cells	S	*n* = 30 adjacent normal liver tissue	Weak expression	2 independent observers
CYP39A1 *CYP39A1*	2014	Khenjanta, C. 25556446	Thailand	CCA unspec	30	30% (9/30)	NR	Tumour cells and hepatocytes	S	*n* = 30, adjacent normal tissue	Weak staining	2 blinded independent observers
DKK1 *DKK1*	2013	Shi, R.Y. 23132676	China	iCCA	138	-	38.4 %	Cytoplasm	TMA	NR	NR	2 observers, 1 blinded
DKK1 *DKK1*	2016	Shi, X.-D. 27608843	China	Hilar CCA	37	64.9%	NR	Cytoplasm	S	Peritumoral tissue	Very low expression	Two blinded independent

												observers
EGFR *ERBB1*	2007	Schmitz 18161916	No, Germany	iCCA	61	21.3% 13/61	68.9%	Tumour cell membrane	S	None	NR	2 blinded observers
EGFR *ERBB1*	2014	Simbolo 24867389	No, Italy	iCCA eCCA	57 40	NR	49% 18%	Membranous	TMA	None	NR	NR
EGFR *ERBB1*	2017	Padthaisong, S. 28668850	Thailand	CCA	94	55%	-	NR	S	NR	NR	NR
EGFR *ERBB1*	2019	Zhao, L. 31307551	China	iCCA	47	-	76.6%	NR	S	NR	NR	1 blinded pathologist
EGFR *ERBB1*	2011	Miyamoto	Japan	iCCA	111	-	26.1%	NR	TMA	NR	NR	3 blinded observers
EGFR *ERBB1*	2010	Pignochino, Y. 21087480	No, Italy	iCCA eCCA	17 19	76.5% 26.3%	100% 52.6%	Membrane	S	Normal cholangiocyte and hepatocyte membranes	All positive	NR
EGFR *ERBB1*	2006	Ogo, Y. 16739351	Japan	CCA unspec	44	43%	-	Membrane	S	+ control Lung Ca, no normal tissues	+ lung Ca	NR
EGFR *ERBB1*	2018	Xu, Y.F. 30177842	China	pCCA	121	45.5%	-	NR	S	NR	NR	NR
EGFR *ERBB1*	2018	Gomes, R.V. 29995151	No, Brazil	CCA (inc pCCA)	35	-	28.6%	Cell membrane	S	NR	NR	1 blinded experienced pathologist
EGFR *ERBB1*	2010	Shafizadeh, N. 20040392	No, USA	iCCA eCCA	26 19	-	81% 79%	Membrane	S	NR	NR	NR

EGFR *ERBB1*	2014	Yang, X. 24927194	China	iCCA eCCA	65 110	30.8% (20/65) 20.9% (23/110)	NR	Membrane	TMA	NR	NR	2 blinded indepen dent observer s
EGFR *ERBB1*	2008	Yoshikawa, D. 18087285	Japan	iCCA eCCA	106 130	NR	27.4 % 19.2 %	Membrane	S	None	NR	3 Blinded authors
EGFR *ERBB1*	2012	Gu, M.J. 22281980	Korea	iCCA	83	NR	55.4 %	Membrane	S	None	NR	NR (althoug h no access to full article)
EGFR *ERBB1*	2016	Moon, A. 27020207	Korea	eCCA	84	NR	38.1 %	Cytoplasm Membrane	TMA	NR	NR	Two authors
EGFR *ERBB1*	2009	Iguchi, T. 19262069	Japan	iCCA	61	NR	67.2 %	Cytoplasm, membranous	S	None	NR	NR
eNOS *NOS3*	2017	Suksawat, M. 27143607	Thailand	CCA	171	100%	50.9 %	Epithelial cells	S	Normal BD	NR	NR
ERα *ESR1*	2018	Kaewlert, W. 30284180	Thailand	CCA	74	78%	-	Cytoplasm and Nucleus	S	Adjacent normal tissue 1. BD epithelial cells 2. Hepatocytes	1. Sig lower 2. High expression in cytoplasm	NR
ERβ *ESR2*	2018	Kaewlert, W. 30284180	Thailand	CCA	74	50%	-	Cytoplasm and Nucleus	S	Adjacent normal tissue 1. BD epithelial cells 2. Hepatocytes	1. Sig lower 2. High expression in cytoplasm	NR
Etk/Bmx *BMX*	2008	Guo, L. 18270973	China	iCCA	57	-	33.3 %	Cytoplasm and nucleus	S	Background liver tissue (normal hepatocytes, BD)	Negative	NR
FOXC2 *FOXC2*	2013	Watanabe, A.	Thailand	eCCA	77	-	23.3 %	Cytoplasm	S	NR	NR	2 indepen

		23919841										dent observers
GLUT-1 SLC2A1	2018	Ikeno, Y. 29642912	Japan	iCCA	50	52%	-	Tumour cell memb	S	NR	NR	2 blinded researchers
GLUT-1 *SLC2A1*	2014	Kubo, Y. 24824030	Japan	iCCA	149	NR	46.3%	Membrane	S	NR	NR	Two blinded authors
HES1 *HES1*	2016	Aoki, S. 27821106	Japan	eCCA	132	Unknown	81.8%	Nucleus	TMA	N = 8 normal BD	Normal BD negative	NR
Hes1 *Hes1*	2013	Gandou, C. 23134772	Japan	pCCA<br>iCCA	25<br>21	-	84%<br>62%	Nucleus, cytoplasm	S	Intra-hepatic and extra hepatic BD	Infrequently positive	NR
Hes1 *HES1*	2012	Igarashi, S. 22594685	Japan	pCCA	43	NR	35%	NR	S	*n* = 28 normal foetal and adult BD	57% (16/28) Hes1 expression in the Nucleus of lining epithelial	NR
ID2 *ID2*	2013	Harder, J. 24409060	No, Germany	iCCA<br>eCCA	50<br>45	-	29%<br>28%	Mainly cytoplasm	S	Normal BD	Low or negative	2 independent blinded observers
IGF-1 *IGF1*	2012	Ohashi, H. 22044563	Japan	eCCA	30	-	60%	NR	S	NR	NR	NR
IGF-2 *IGF2*	2012	Ohashi, H. 22044563	Japan	eCCA	30	-	53%	NR	S	NR	NR	NR
IGFBP3 *IGFBP3*	2006	Jinawath, N. 17006947	Thailand	iCCA	273	NR	55.34%	Cytoplasm	TMA	Noncancerous liver tissue from *n* = 10 patients with liver metastasis from CRC	Negative expression	Three independent investigators

IL-17<br>IL17	2015	Asukai, K.<br>26228109	Japan	iCCA	72	38.9%	-	Cytoplasm membrane	S	Pancreas exocrine tissue +ve control. Peritumoral normal liver	Pancreas +ve, normal liver	NR
IL-6<br>IL6	2015	Asukai, K.<br>26228109	Japan	iCCA	72	47%	-	Cytoplasm membrane	S	Appendix +ve control	Appendix +ve	NR
IMP3<br>*IMP3*	2014	Gao, Y.<br>24745619	China	iCCA	72	-	82%	Tumour nests	S	Adjacent normal tissue	No expression	NR
IMP3<br>*IMP3*	2009	Riener, M.-O.<br>19467694	No, Switzerland	iCCA<br>eCCA	19<br>58	-	36.8%<br>50.0%	Cytoplasm	TMA	Normal BD (n = 36), acute inflammation (n = 36), dysplasia (low grade n = 9, high grade n = 11)	Normal BD, inflammation, low grade dysplasia negative/weak, high grade all +	3 pathologists
IMP3<br>*IMP3*	2014	Lok, T.<br>24439226	China	iCCA	41	68%	90%	Cytoplasm	S	NR	NR	3 investigators
IMP3<br>*IMP3*	2012	Chen, Y.-L.<br>23246869	Taiwan	iCCA	61	-	41.0%	Tumour cells	S	Adjacent normal/ non tumorous liver tissue	Negative	2 blinded independent pathologists
IMP3<br>*IMP3*	2013	Kawashima, H.<br>23269460	Japan	eCCA	68	NR	74.9%	Cytoplasm	S	n = 12 benign	Detected in 1/12 samples	Two independent observes
Laminin 5<br>*LAMA5*	2018	Soejima, Y.<br>29584696	Japan	iCCA	48	54.2% (26/48)	NR	Cytoplasm of tumour cells and intratumoral stroma	S	None	NR	NR
LEF1<br>*LEF1*	2012	Coulouarn, C.<br>22696594	France	CCA	54	NR	32%	Nucleus	TMA	Normal biliary cells	Weak expression?	NR

LI-cadherin<br>*CDH17*	2009	Takamura, M.<br>23852458	Japan	iCCA	34	NR	52.9 %	NR	S	Non-neoplastic biliary epithelium negative	Non-neoplastic biliary epithelium negative	NR
Lipocalin 1<br>*LCN1*	2018	Tian, Y.<br>AN: 624752303	China	iCCA<br>eCCA	100<br>27	44.1%	-	NR	TMA	Adjacent non-tumour liver tissue (*n* = unknown)	CC > than adjacent normal tissues	2 independent blinded pathologists
MAGE-A1<br>*MAGEA1*	2011	Zhou, J.X.<br>21211023	China	iCCA	89	-	29.2 %	Cytoplasm	S	NR	NR	NR
MAGE-A3/4<br>*MAGEA3*<br>*MAGEA4*	2011	Zhou, J.X.<br>21211023	China	iCCA	89	-	27.1 %	Cytoplasm	S	NR	NR	NR
Matripase<br>*ST14*	2019	Komatsubar, A.T.<br>30729623	Japan	eCCA	256	Cells 48%<br>Stroma 40%	-	Mainly cytoplasm	TMA	Non-malignant epithelial cells	Matriptase + in some tumors	2 blinded indep pathologists
M-CSF<br>*CSF1*	2015	Oishi<br>25052379	Japan	iCCA	39	NR	35.9 %	Cytoplasm	S	Adjacent hepatic paranchyma	None	Single blinded pathologist
Mesothilin<br>*MSLN*	2012	Kawamata, F.<br>23064529	Japan	eCCA	61	47.5%	72%	luminal membrane/ cytoplasm	S	NR	NR	3 independent blinded pathologists
MMP7<br>*MMP7*	2009	Oka, T.<br>19701966	Japan	CCA	30	NR	80%	Cytoplasm	S	None	NR	NR
MMP7<br>*MMP7*	2011	Ohashi, H.<br>22044563	Japan	eCCA	30	-	80%	NR	S	NR	NR	NR

MMP7 *MMP7*	2016	Shi, X.-D. 27608843	China	pCCA	37	62.2%	NR	Cytoplasm	S	Peritumoral tissue	Very low expression	Two blinded indepen dent observer s
MMP9 *MMP9*	2018	Park, Y. 29422250	Korea	eCCA	66	50%	-	NR	S	NR	NR	NR
MMP9 *MMP9*	2014	Sun, Q. 25337264	China	pCCA	58	46.6%	67.2 %	Cytoplasm	S	NR	NR	2 indepen dent blinded observer s
MMP9 *MMP9*	2009	Itatsu, K. 19218340	Japan	eCCA iCCA	75 35	NR	58%	Cytoplasm	S	Adjacent normal non-neoplastic BD	Faint or absent expression	NR
MMP9 *MMP9*	2015	Tian, X. 24985031	China	iCCA	65	-	53.8 %	Cytoplasm	S	NR	NR	2 blinded indepen dent observer s
MMP9 *MMP9*	2010	Subimerb	Thailand	iCCA eCCA	32 18	Overall 6% CCA cells, 44% of TAMs in invasive margin	- -	CCA cells Tumour associated macrophages	S	No control	NR	NR
MMP9 *MMP9*	2015	Sun, Q. 26339407	China	pCCA	62	32.2%	67.7 %	Cytoplasm	S	NR	NR	2 indepen dent blinded observer s
MMP9 *MMP9*	2013	Shi, R.Y. 23132676	China	iCCA	138	-	45.6 %	Cytoplasm	TMA	NR	NR	2 observer

MMP9<br>*MMP9*	2009	Onodera, M.<br>19721413	Japan	eCCA<br>iCCA	55<br>32	-	47.3 %<br>62.5 %	Cytoplasm	S	Non-neoplastic biliary epithelium (*n* = 13)	Negative	s, 1 blinded 2 independent observers
Nanog<br>*NANOG*	2019	Zhang, M.-X.<br>30854141	China	iCCA	116	61.2%	-	Cytoplasm	TMA	Para-carcinoma tissues	Weak/ negative	2 experienced observers
NGF<br>*NGF*	2014	Yang, X.-Q.<br>24744599	China	iCCA	83	27.7% (23/83)	NR	Cytoplasm	S	None	NR	NR
Notch4<br>*Notch4*	2014	Wu, W.-R.<br>25031748	China	iCCA	41	NR	34.1 %	Nucleus and cytoplasm	S	Adjacent non-tumour liver	Expressed in normal liver tissue	NR
NY-ESO-1<br>*CTAG1B*	2011	Zhou, J.X.<br>21211023	China	iCCA	89	-	21.3 %	Cytoplasm	S	NR	NR	NR
OPCML<br>*OPCML*	2011	Sriraksa, R.<br>21448164	Thailand	CCA	92	NR	26%	NR	S	*n* = 29 tumour adjacent normal samples	NR	Two independent observers
p53<br>*TP53*	2006	Hughes, N.R.<br>16679351	Australia & Thailand	iCCA<br>eCCA<br>iCCA<br>eCCA	27<br>38<br>13<br>34	44%<br>47%<br>15%<br>26%	+ve only if strong staining of most cells	Nucleus	S	Normal donor livers *n* = 6, non-neoplastic biliary epithelium	Normal bile ducts and liver negative	NR

p53 *TP53*	2018	Kim, S.J. 29309301	Korea	iCCA	213	NR	43.7 %	Nucleus	TMA	None	NR	NR
p53 *TP53*	2009	Shen, Y.C. 19190145	Taiwan	iCCA eCCA	74 49	NR	27% 36%	Nucleus	S	NR	NR	NR
p53 *TP53*	2019	Zhao, L. 31307551	China	iCCA	47	-	31.9 %	NR	S	NR	NR	NR
p53 *TP53*	2017	Okada, R. 28508195	Japan	eCCA	61	34.4%	-	Nucleus	S	NR	NR	2 observer s
p53 *TP53*	2012	Tan, X.-P. 22783387	China	CCA	69	-	84%	Nucleus	S	No control	NR	2 blinded indepen dent patholog ists
p53 *TP53*	2017	Liu, S. 28915621	China	iCCA	60	-	46.7 %	NR	S	*n* = 60 paired normal tissue	NR	NR
p53 *TP53*	2011	Ohashi, H. 22044563	Japan	eCCA	30	-	46.7 %	NR	S	NR	NR	NR
p53 *TP53*	2010	Riener, M.-O. 20621328	Switzerlan d	iCCA eCCA	19 59	NR	37% 46%	Nucleus	TMA	*n* = 30 normal biliary epithelium of extrahepatic BD	Negative for p53 expression	NR
p53 *TP53*	2008	Karamitopo ulou, E. 18854271	Switzerlan d	eCCA	62	NR	32%	Nucleus staining	TMA	*n* = 24 normal BD	Not expressed in normal tissue	NR
p53 *TP53*	2013	Kaira, K. 24131658	Japan	eCCA	89	49.4% (44/89)	NR	Nucleus	S	*n* = 16 benign biliary track lesions	Not expressed in control tissue 0%	2 blinded authors
p53 *TP53*	2010	Won, H.S. 20955617	Korea	eCCA inc. pCCA	75	NR	53.3 %	Nucleus	TMA	None	NR	Two blinded indepen dent observer s
p53 *TP53*	2006	Liu, X.-F. 16937443	China	CCA	36	NR	52.8 %	Nucleus	S	None	NR	NR

p53 *TP53*	2013	Hirashima, K. 22927259	Japan	eCCA	71	NR	39.4 %	Nucleus	S	Normal BD epithelium of the samples	NR	Two blinded authors
p53 *TP53*	2015	Vasuri, F. 25367684	Italy	iCCA	60	NR	38.3 %	Nucleus	TMA	*n* = 10 normal liver tissue	NR	NR
p53 *TP53*	2009	Iguchi, T. 19262069	Japan	iCCA	61	NR	36.1 %	Nucleus	S	None	NR	NR
p53 *TP53*	2015	Keira, Y. 25503275	Japan	iCCA eCCA	27 32	NR	NR	Nucleus of adenocarcinomas	S	NR	NR	NR
p53 TP53	2016	Moro, C.F. 27829056	No, Sweeden	iCCA	97	-	30%	Nucleus	S	NR	NR	2 pathologists
p53 TP53	2017	Bhalla, A. 28823571	No, USA	iCCA	56	-	55.4 %	Nucleus	S	Liver with Von Meyenburg complex without dysplasia and with low grade dysplasia	No p53 expression at 10% cut off	NR
p53 *TP53*	2014	Lee, C.T. 25245603	China	iCCA	54	-	59%	Nucleus	S	NR	NR	2 pathologists
p53 *TP53*	2013	Park, K.W. 23613672	Korea	eCCA pCCA iCCA	46 41 17	-	52.8 % 47.4 % 46.2 %	Nucleus	TMA	NR	NR	2 blinded independent pathologists
PAI-2 *SERPINB2*	2018	Utaijaratrasmi, P. 29347950	Thailand	iCCA	72	63%	-	Tumour cell	S	NR	NR	2 blinded independent observers

PBK/TOPK *PBK*	2010	He, F. 19954816	China	iCCA pCCA	51 23	-	91.9 %	Cytoplasm, occ membranous	S	N = 10 normal donor livers	Expressed in N BD but -ve in hepatocytes	NR
PDGF-D *PDGFD*	2019	Komatsubara, T. 30729623	Japan	eCCA	256	Cells 32% Stroma 26%	-	Mainly cytoplasm	TMA	Adjacent non-malignant epithelial cells	weak or no staining of PDGF-D	2 blinded indep pathologists
PGF *PGF*	2006	Jinawath, N. 17006947	Thailand	iCCA	273	NR	70.8 3%	Cytoplasm	TMA	Noncancerous liver tissue from *n* = 10	NR	Three independent investigators
PIWIL2 *PIWIL2*	2015	Chen, Y.J. 26125915	China	pCCA	41	80.5%	-	Cytoplasm	S	Control tissues *n* = 10	90% low or negative	NR
PRL-2 *PTP4A3*	2010	Xu, Y. 19757198	China	iCCA	102	47.1%	-	Cancer cells	S	Adjcent non-cancerous liver *n* = 102	Negative or low	All sections scored twice
ROS1 *ROS1*	2016	27136744	Taiwan	iCCA	85	-	73%	Cytoplasm	S	NR	NR	2 blinded independent pathologists
ROS1 *ROS1*	2015	Lee, K.-H. 26475437	Korea	iCCA	194	NR	37.1 %	Cytoplasm	TMA	Each TMA had 4 cores of normal tissue	NR	NR
SALL4 *SALL4*	2015	Deng, G. 26317546	China	iCCA	175	-	58%	Nucleus	S	Adjacent non-tumour tissue	N = 28 negative	3 experienced pathologists
SFRP1 *SFRP1*	2016	Davaadorj 28062160	Japan	iCCA	50	NR	60%	NR	S	None	NR	Single blinded

sFRP1 *SFRP1*	2014	Kang, P. 24594839	China	iCCA eCCA	20 58	-	30% 34.5 %	Cytoplasm	S	Normal BD n = 36	69.4%	patholog ist 2 blinded indepen dent observer s
SHH *SHH*	2013	Tang, L. 23493353	China	iCCA	108	-	93.5 %	Cytoplasm	TMA	Normal liver n = 8	Normal BD - ve/weak	NR
SHH *SHH*	2012	Kim, Y.J. 21946948	Korea	eCCA	42	38.1%	85.7 %	Cytoplasm	S	Paired normal liver n = 42	35.7% normal liver positive	NR
TBX4 *TBX4*	2013	Zong, M. 23341740	China	iCCA	72	-	52.8 %	Nucleus, occ cytoplasm	S	Paired adjacent non-cancerous tissue	2.78% positive	NR
TCF7 *TCF7*	2019	Liu, Z. 31248836	China	pCCA	103 + 57	-	43.8 %	NR	TMA	No control	NR	NR
Tenascin *TNC*	2009	Iguchi, T. 19262069	Japan	iCCA	61	NR	63.9 %	Intra-tumoral stroma	S	None	NR	NR
Tenascin-C *TNC*	2018	Soejima, Y. 29584696	Japan	iCCA	48	37.5% (18/48)	NR	Cytoplasm of tumour cells and intratumoral stroma	S	None	NR	NR
TFF1 *TFF1*	2007	Thuwajit, P. 17397518	Thailand	CCA	61	91.8%	98.4 %	Cytoplasm, apical biliary epithelium, lumen of BD, stroma	S	Adjcent normal liver	+ large BD but negative hepatocytes and small BD	NR
TFF1 *TFF1*	2008	Chiu, C.-T. 18329969	Taiwan	iCCA	30	NR	56%	NR	S	None	NR	NR
TFF1 *TFF1*	2006	Jinawath, N. 17006947	Thailand	iCCA	273	NR	28.5 7%	Cytoplasm	TMA	Noncancerous liver tissue from n = 10	NR	Three indepen dent

												investigators
TFF1 *TFF1*	2018	Kaewlert, W. 30284180	Thailand	CCA	74	53%	-	Cytoplasm	S	Adjacent normal BD epithelial cells	Negative	NR
TFF2 *TFF2*	2008	Thuwajit, P. 18410610	Thailand	CCA	51	31% (16/51)	NR	Cytoplasm and lumen	S	NR	NR	Two blinded observers
TGF beta 1 *TGFB1*	2015	Chen, Y. 25993985	China	iCCA	78	29.5%	47.4 %	Cytoplasm	S	Normal liver tissue	Hepatocytes and BD -ve	NR
TGFβ1 *TGFB1*	2018	Soejima, Y. 29584696	Japan	iCCA	48	45.8% (22/48)	NR	Cytoplasm	S	None	NR	NR
Tie2 *TEK*	2008	Mihara, Y. -	Japan	eCCA	119	-	774.8%	Cytoplasm	S	Normal mucosa n = 38	23.7%	2 independent investigators
TLR2 *TLR2*	2016	Liu, B. 27616304	China	iCCA	51	47%	-	NR	S	Paired normal n = 51	Significantly lower	NR
Transferrin *TF*	2017	Jamnongkan, W. 28671021	Thailand	CCA	49	41%	-	Cytoplasm	S	Cadaveric liver donors n = 3	Lower in N BD cells compared to CCA	NR
Twist *TWIST1*	2014	Nitta, T. 25077440	Japan	eCCA	117	57.3%	-	NR	TMA	NR	NR	2 blinded investigators
Twist *TWIST1*	2016	Chu, C. 26479153	China	CCA	54	-	27.7 %	NR	S	Choledochal cyst (n = 16)	87.5% positive	Single senior pathologist
Twist *TWIST1*	2014	Duangkumpha, K. 24969562	Thailand	CCA	30	30% (9/30)	NR	Nucleus and cytoplasm of tumour cells	S	None	NR	NR

Type IV Collagen a6 *COL4A6*	2013	Hirashima, K. 22927259	Japan	eCCA	71	NR	38%	Membrane	S	Normal BD epithelium of the samples	Weak expression	Two blinded authors
ULBP1 ULBP1	2016	Tsukagoshi, M. 26608587	Japan	eCCA	82	51.2%	100%	Tumour cells	S	NR	NR	NR
ULBP2 ULBP2	2016	Tsukagoshi, M. 26608587	Japan	eCCA	82	61.0%	76.8%	Tumour cells	S	NR	NR	NR
uPA *PLAU*	2012	Thummarati, P. 22294827	Thailand	CCA	174	49.4%	75.3%	Cytoplasm	TMA	NR	NR	NR
VEGF C *VEGFC*	2008	Aishima 18192971	Japan	iCCA	88	NR	30%	Cytoplasm	S	None	NR	Two blinded pathologists
VEGF C	2006	Taniguchi, K. 16688800	Japan	pCCA	34	-	47%	Cancer cells	S	NR	NR	Single blinded pathologist
VEGF C *VEGFC*	2015	Xu, Y.F. 25805932	China	iCCA	65	-	49.2%	Cytoplasm	TMA	NR	NR	2 independent blinded observers
VEGFC *VEGFC*	2017	Suksawat, M. 27143607	Thailand	CCA	171	100%	66.7%	Cytoplasm and membrane	S	No control	NR	NR
VEGF-C *VEGFC*	2006	Park, B.K. 16601431	Korea	iCCA	36	41.7%	69.5%	Cytoplasm	S	Normal epithelia	Negative	2 blinded observers
VEGF-C *VEGFC*	2015	Zhao, R. 26622616	China	CCA	65	NR	75.4%	NR	S	n = 5 normal cholangiolar biopsies	Moderate to low in 5% of BD	NR
VEGF-C *VEGFC*	2013	Shi, R.Y. 23132676	China	iCCA	138	-	72.5%	Cytoplasm	TMA	NR	NR	2 observer

												s, 1 blinded
VitD R VDR	2007	Seubwai, W. 17487855	Thailand	CCA	111	40%	74%	Cytoplasm	S	Normal BD	20% low expression	NR
Wnt3 *WNT3*	2014	Loilome, W. 24549785	Thailand	CCA	38	Unknown	42.1 %	Tumour cells	S	Cadaveric donor liver. Adjacent non-tumour tissue	Normal BD & hepatocytes -ve.	NR
Wnt3a *WNT3A*	2014	Loilome, W. 24549785	Thailand	CCA	38	Unknown	92.1 %	Cytoplasm	S	Cadaveric donor liver. Adjacent non-tumour tissue	Normal BD low expression.	NR
Wnt5a *WNT5A*	2014	Loilome, W. 24549785	Thailand	CCA	38	Unknown	76.3 %	Tumour cells	S	Cadaveric donor liver. Adjacent non-tumour tissue	Normal BD epithelia weak cytoplasm +ve.	NR
Wnt7b *WNT7B*	2014	Loilome, W. 24549785	Thailand	CCA	38	34.2%	100 %	Cytoplasm	S	Cadaveric donor liver. Adjacent non-tumour tissue	Nucleus +ve hyperplastic BD. +ve	NR
ZNF423 *ZNF423*	2019	Chaiprasert, T. 31284679	Thailand	iCCA	75	41%	-	NR	S	Adjacent non-cancer areas	NR	NR

© 2020 by the authors. Licensee MDPI, Basel, Switzerland. This article is an open access article distributed under the terms and conditions of the Creative Commons Attribution (CC BY) license (http://creativecommons.org/licenses/by/4.0/).
